# Effects of Temperature and Tempo: Evaluating How Much Time in a Typical Community-Based Yoga Class Is Moderate-Intensity Aerobic Activity

**DOI:** 10.3390/ijerph20032349

**Published:** 2023-01-28

**Authors:** Anna Dysart, Samantha M. Harden

**Affiliations:** Department of Human Nutrition, Foods, and Exercise, Virginia Tech, Blacksburg, VA 24061, USA

**Keywords:** yoga, physical activity, physical activity guidelines

## Abstract

The 2nd edition of the Physical Activity Guidelines for Americans (PAG) recommends, in part, 150 min of moderate-intensity aerobic physical activity. The PAG states that yoga may be able to help meet the guidelines for moderate-intensity aerobic exercise for adults and older adults. Our study aims to objectively measure the proportion of time participants’ activity that is categorized as moderate-intensity aerobic activity based on heart rate data and to subjectively measure rate of perceived exertion (RPE). Participants completed the Stanford Leisure-Time Activity Categorical item to establish baseline PAG aerobic activity compliance. Participants then completed four separate 1-h yoga sessions at different tempos (cadence) and temperatures while wearing heart rate monitors. During and directly after the session they also marked their RPE on a modified 10-point scale. All participants reached moderate-intensity aerobic activity for at least some portion of a yoga session based on heart rate monitor data and RPE. The average duration of moderate intensity was 32.75% of the class across all four class types, with no significant differences by condition. Age was a significant factor in time spent in moderate-to-vigorous physical activity for only the thermo-neutral Hatha classes (*p* = 0.010). Tempo, temperature, and baseline activity levels were not significant factors in time spent in moderate-to-vigorous intensity physical activity based on Pearson Correlation and the Kruskal–Wallis test. Hatha and Vinyasa yoga classes, at room or hot temperatures, can be used to meet a portion of the PAG moderate-intensity activity recommendations.

## 1. Introduction

The 2nd edition of the Physical Activity Guidelines for Americans (PAG) recommends 150 min of moderate-intensity aerobic physical activity and two days of muscle strengthening each week to promote health for those 18 years of age and older [1]. In addition, balance practices are recommended for those 65 years of age and older. Approximately 25% of adults are meeting the current PAG [2]. Yoga is a multicomponent activity that includes balance, muscle strengthening, and aerobic components [1,3] and has been associated with positive psychological and physiological benefits [4,5,6,7,8,9,10,11]. When yoga was compared with physical activity, as pertaining to health-related quality of life, a systematic review and meta-analysis by Patel et al. found that yoga was superior to traditional forms of physical activity (e.g., walking, running) for older adults [12]. The most recent Physical Activity Guidelines Advisory Committee Scientific Report [13] suggested further studies regarding the benefits of yoga for adults.

Some of the reasons the PAG was unable to conclude that all yoga can help meet the recommendations include (1) the practice of yoga has a variety of different aspects and meanings based on the lineage from which the yoga practitioner comes [5,9,11]; (2) few studies have been conducted on yoga and its relative intensity, or ability to meet the PAG [14]; (3) existing studies have focused on young, healthy participants in metropolitan areas who are fairly experienced with yoga [14]; and (4) research designs have yet to parse out mechanisms of the exercise impact of yoga versus the temperature of the room or experience level affecting heart rate. Notably, a reductionist approach to the physical activity component of yoga is controversial, as yoga is a multifaceted mind–body practice with ancient roots related to yuj (union) with self and the divine. Yoga, as a spiritual and cultural practice, has never been about physical mastery of postures [15], so much research has been done regarding stress reduction [5,11,16], worry and anxiety [10], but less research has specifically looked at yoga as a way to get aerobic physical activity in addition to its other benefits. Furthermore, data collection on older adults, as well as those who may be less experienced with yoga, and the PAG has been limited [14]. While yoga is not solely a physical practice, being able to meet some of the PAG through yoga might help engage public interest in yoga, especially for older adults. 

While some research suggests that yoga does not reach moderate aerobic activity [3,14,16], weaknesses in these studies relate to the short duration of the sessions (15–20 min compared with 60-min classes typically available in studios) [3,14,16], as well as the fact that the sessions were led by video (which might lead to less engaged participation)^3^ and, finally, the postures were repetitive and included a number of supine and seated postures [3,14,17]. A yoga study with 6 weekly high-intensity yoga sessions (30–40 min of sun salutations followed by 15 min of other poses) with ‘additional home training’ found no improvement in cardiovascular fitness when tested through a Cooper running test [18]. The rate of perceived exertion (RPE) during sessions was on average 14, which is within the cardiovascular training range, but heart rate was not monitored during these study sessions [18]. 

In contrast, and in favor of the potential of yoga classes for meeting moderate to vigorous physical activity (MVPA), multiple studies that had more dynamic flows (sequences) and longer sessions found improvements in a 6-min walk test [19] and heart rate and exertion [20]. A study looking at power yoga, heart rate, skin temperature, and body mass determined that power yoga could be considered a moderate–vigorous-intensity activity with 45-min sessions [21]. 

Despite these existing studies and calls to action, there is a gap in the research on understanding the intensity of exercise offered within a typical yoga studio including “flow,” Hatha/classical, and hot yoga. Most community yoga classes are about 60 min in length [3,14], and yoga classes of that length and in a more pragmatic setting have not been thoroughly investigated [14]. There is also minimal research performed on whether wearable activity trackers and accelerometers are able to accurately assess heart rate or movement, and therefore activity intensity level, in a yoga setting. Making a connection between yoga participants of all ages and backgrounds and common fitness tracking technology is important to help a broader population feel empowered to engage in more physical activity.

Therefore, the purpose of this study was to test four hypotheses: a typical yoga class offered in a community setting will get participants into a moderate heart rate zone for at least some portion of the class (H1); individuals who are not meeting PAG aerobic recommendations at baseline will have a higher heart rate during some of the yoga asana (physical yoga practice) (H2); warmer temperatures will result in higher RPE (H3); and faster tempos will result in higher reported RPE (H4).

## 2. Materials and Methods

### 2.1. Recruitment

Subjects were primarily recruited through a local yoga studio (In Balance Yoga) in Blacksburg, Virginia, via posted flyers, class announcements, and the studio newsletter. Recruitment was also conducted over Twitter and email listservs. Approved flyers were posted on the Virginia Tech campus and at local community centers, faith-based organizations, and gathering places. Participants completed the Physical Activity Readiness Questionnaire Plus (PAR-Q+) [22] as a screening tool to join the study, and if the PAR-Q+ score indicated that it was safe for them to participate in physical activity, they were sent the consent form and survey. If the PAR-Q+ indicated a need for further screening, the ePARmed-X+ [23], which is an additional, more detailed screening tool for physical activity, was completed. If they were not indicated as safe for physical activity based on their responses to the PAR-Q+ and/or ePARmed-X+, they were not eligible for the study and were informed of their noneligibility. 

### 2.2. Orientation and Equipment

Participants attended an orientation session via Zoom that detailed information and study protocols for participants. This included a brief overview of how to wear a heart rate monitor and the foundations of the asanas (postures). The recording was available for those who could not attend live via Zoom. They were randomly assigned to intervention arms (see Table 1) and then informed of their times/days when they picked up their equipment. Participants were given Polar Verity Sense heart rate monitors based on previous yoga studies and validation [21], The Polar Verity Sense is the newer version of the Polar OH1, with increased Bluetooth range and longer battery life. 

### 2.3. Study Interventions

Participants were requested to complete a survey on their current activity level (utilizing the Stanford Leisure-Time Activity Categorical (L-CAT) item) [24] and familiarity with yoga (utilizing the Yoga Self-Efficacy Scale) [25]. 

The yoga sequence for each class was standardized between sessions and crafted by two registered yoga teachers to match a typical yoga class, containing breathing practice, warm-up, conditioning, and cool-down postures with many of the same postures utilized in previous studies [3,21] (see Appendix A: Table A1). Yoga sessions were led by the same 500-h registered yoga teacher, wearing the same outfit and using the same playlist. This reduces the risk for injury due to the extensive training the yoga teacher has received and his or her knowledge of cuing and sequencing for best practice while attempting to control as many implementation factors as possible. The Hatha classes held each pose for five breaths while the flow class held approximately one breath per movement (i.e., continuous flow from posture to posture) and repeated the sequence twice. Sessions were Hatha thermo-neutral, flow thermo-neutral, Hatha hot, and flow hot yoga (see Table 1). During the 60-min yoga sessions, participants and the instructor wore the heart rate monitors. 

To assess during- and after-class rate of perceived exertion (RPE), participants were asked to rate their exertion level (on a sheet of paper that was placed by their mat) using a scale of 1–10, with 1 being ‘very light activity’ to 10 being ‘maximum effort’. The modified version of the original Borg RPE has been shown to be highly correlated to the original and still shows exercise intensity [26], while also possibly being more familiar to participants who have seen it used by group fitness personnel [27] or in the medical field [28].

### 2.4. Statistical Analysis

After all four sessions were completed, data from the heart rate monitors was assessed using SPSS, version 26 (IBM, Chicago, IL, USA) to determine the time each participant spent in each intensity zone, based on their age-predicted max heart rate using the adjusted Tanaka, et al., equation (208 − 0.7 × age) [29]. Each intensity zone was defined based on the following definitions: light <60% maximum heart rate, moderate 60–75% maximum heart rate, vigorous 76–90% maximum heart rate, and near maximal >90% maximum heart rate [1]. The Kruskal–Wallis test was used to determine differences in sessions.

The RPE of each class was assessed by averaging the participants’ answers and using independent t-tests to examine the differences between age groups. The RPE of each participant was also compared to the data from their heart rate monitor for differences in perceived exertion versus heart rate. 

## 3. Results

### 3.1. Sample

A total of 21 participants (9 of whom (43%) were 65+ years of age) were recruited for the study. The mean age was 48.1 ± 21.82 years with a range of 19–74 years. Sixteen participants completed all 4 types classes, with the other 5 participants completing 2–3 of the 4 classes. Due to unforeseen issues with the heart rate monitors, data were collected from 9 participants for the hot Vinyasa–style classes, 11 participants for the room-temperature Vinyasa and hot Hatha classes, and 14 participants for the room-temperature Hatha classes. Only 3 participants had personal wearable activity trackers. Data were obtained from all 3 for the room-temperature Hatha class, from 2 participants for the room-temperature Vinyasa and hot Vinyasa classes, and from 1 participant for the hot Hatha class. Further demographic information is in Table 2. 

### 3.2. MVPA

**Figure 1:**
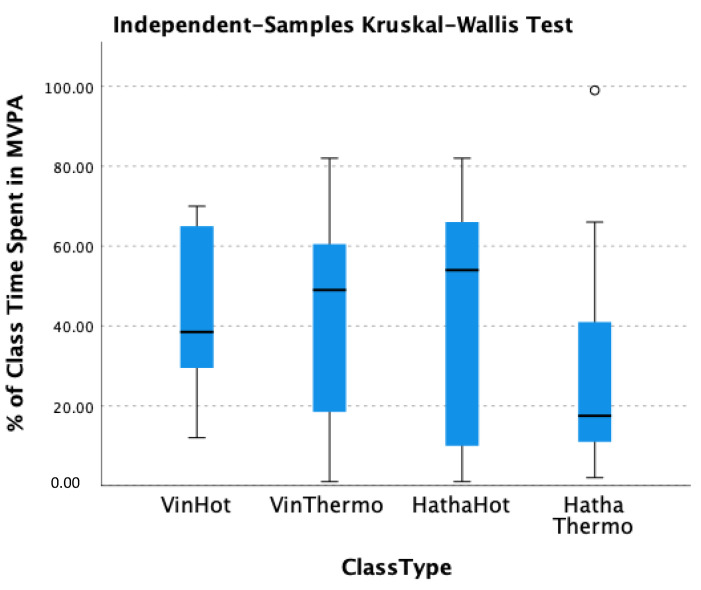
Kruskal–Wallis Box Plot for Differences Between Classes. Outlier denoted by °.

When looking at all participants and all class types on the basis of heart rate data, there was an average of 32.75% MVPA within the approximately 60-min classes. By condition, this was 38.57% (±24.54%) for Vinyasa hot, 34.45% (±32.67%) for Hatha hot, 40.55% (±29.09%) for Vinyasa thermo-neutral, and 24.93% (±28.63%) for Hatha thermo-neutral. Notably, 2 participants did not meet MVPA for any duration of the room-temperature Hatha class, and only 1 participant did not meet MVPA at any time during the other three class types. However, every participant reached MVPA in at least one of the classes. Using the independent samples t-test, age was a significant factor in time spent in MVPA for only the thermo-neutral Hatha classes, in that those who were older were more likely to be in higher heart rate zones (*p* = 0.010). Tempo, temperature, and baseline activity levels were not significant factors in time spent in MVPA based on the Kruskal–Wallis test, see Figure 1. See Table 3 for specific class information.

### 3.3. RPE

The midpoint RPE was on average within the moderate intensity range (5.8 (1.8)), with no significant differences in RPE reports by younger and older adult (*p* > 0.05). For the end-of-class RPE, participants who were over 65 reported significantly higher RPE when compared with their younger counterparts (*p* < 0.05) after every session. 

## 4. Discussion

This study was able to demonstrate among a small number of diverse participants that all individuals achieved a moderate- or vigorous-intensity heart rate zone during at least one of these yoga sessions, regardless of tempo or temperature. However, although not significant, the percent of time in MVPA trended in the hypothesized direction with hotter and faster classes having more time in MVPA than the Hatha thermo-neutral class. This provides preliminary evidence that regardless of age, tempo, temperature, or baseline physical activity level, a typical yoga class can contribute to the 150 min of MVPA recommended per week, especially since the 2nd edition of the PAG removed the requirement for MVPA to be performed in 10-min increments [1].

These data are in agreement with those of other studies [19,20] that were conducted focusing on yoga classes that were more similar to community-based yoga classes (i.e., longer duration [16] and led by a live instructor). These heart rate data are also in agreement with the subjective RPE data that were, on average, in the moderate-intensity range of perceived exertion. More research utilizing both RPE data and data from other activity trackers commonly found in community-based yoga classes, such as FitBits and Apple Watches, is needed. 

Additionally, many participants choose hot and/or flow classes when a studio offers a variety of classes [6]. Unlike in previous studies [13], the participants recruited for our study had a variety of levels of experience with yoga, many being new to the practice. This is also more similar to a community-based yoga class that participants might attend with a mixture of regular yoga practitioners and nonregular practitioners. However, in a study that looked at Bikram yoga, a hot style yoga, it was found that participants with more yoga experience had higher heart rates [30]. Future studies may look at creating sessions with experienced and novice yoga practitioners together and in separate classes to see how changing the demographics of the class affects participants’ heart rates.

In keeping with the community-based class framework, and varying from the methods of previous studies [3,21], participants were not instructed to avoid physical activity on the days that they were attending sessions. Some participants ran or rode their bike to class, which may have affected their heart rates, particularly during the beginning of class. However, this reflected their normal physical activity levels, as one participant’s only form of transportation was bike riding. Future research looking into the effects of daily physical activity, medicines, and other factors on heart rate during yoga is needed.

Spreading the four classes over 2 weeks helped prevent mental and physical fatigue in the yoga instructor. Additionally, having multiple class styles that each participant attends within the research study is novel in that many previous studies have looked at the energy expenditure or heart rate data of participants after having attended only one yoga session or one style of yoga [3,14,21,30]. Future research into the physical and mental demands of instructing yoga classes would be appropriate, as yoga teachers may instruct multiple classes per day or per week. The spread of classes may have been a negative in getting participants to be available for all four sessions. Participants who missed sessions had their data removed from the between-session comparisons, but the data for each session were also analyzed on a session-by-session basis.

Many previous yoga studies have been limited by lack of demographic diversity [3,14,16]. Care was taken in the recruitment materials and placement of recruitment flyers for this study to be welcoming to participants of all genders and ethnicities. The participant demographics reflect the community from which participants were recruited for this study [7]. Due to equipment and COVID restrictions, cohort sizes were kept small. A larger participant pool might bring in a greater diversity among participants.

## 5. Conclusions

Based on the heart rate and RPE data, the time adults and older adults spend in MVPA within a typical Hatha or Vinyasa yoga class can be helpful in meeting the PAG guidelines, with some participants meeting as much as one-third of the recommended 150 min of MVPA in a single class. While the entirety of the class is unlikely to meet MVPA, knowing that yoga may contribute to reaching the PAG guidelines may encourage the promotion of yoga as exercise by healthcare providers and public health workers. This study will help build the body of evidence for yoga to be used and recommended as a way to help meet the aerobic activity components of the PAG.

## Figures and Tables

**Table 1:** Yoga Class Sessions Descriptors.

	Hatha Thermo-Neutral	Flow Thermo-Neutral	Hatha Hot	Flow Hot
Temperature	78 °F	78 °F	92 °F	92 °F
Cadence	5 breaths per posture	1 breath per posture	5 breaths per posture	1 breath per posture
Set sequence (See Appendix A)	Complete Set 2 twice Complete Set 3 once on each side	Complete Set 3 3 times Complete Set 3 once on each side	Complete Set 3 3 times Complete Set 3 once on each side	Complete Set 2 twiceComplete Set 3 once on each side
Opening pranayama (breath work)	Guided equal parts breathing in standing mountain pose (Sama Vritti in Tadasana) for 2 min			
Savasana	5 min			

Complete flow sequence be found in Appendix A.

**Table 2:** Study participant demographics.

Study Participant Demographics (*n* = 21)	
Age, M(SD)	48.1 (21.82)
Sex, *n* (%) Male Female Declined to answer	7 (33.3) 13 (61.9) 1 (4.8)
Ethnicity, *n* (%) White Black Asian Declined to answer	16 (76.2) 2 (9.5) 2 (9.5) 1 (4.8)
Physical Activity Level, *n* (%) Insufficiently Active Meeting Physical Activity Guidelines Very Active Declined to answer	9 (42.9) 5 (23.8) 6 (28.5) 1 (4.8)
Regular Yoga Practitioner Yes No Declined to answer	8 (38.1) 12 (57.1) 1 (4.8)

**Table 3:** Percent Time in MVPA and RPE Results for Each Class.

	Hatha Thermo-Neutral	Vinyasa Thermo-Neutral	Hatha Hot	Vinyasa Hot
Avg % Time in MVPA (by HR)				
All M(SD)	24.93 (28.63) (*n* = 13)	40.55 (29.09) (*n* = 12)	34.45 (32.67) (*n* = 11)	38.57 (24.54) (*n* = 9)
<65 M(SD)	22.61 (14.56) (*n* = 7)	34.95 (27.11) (*n* = 7)	38.65 (37.55) (*n* = 7)	44.93 (22.33) (*n* = 7)
≥65 M(SD)	31.59 (40.95) (*n* = 6)	40.48 (36.75) (*n* = 5)	24.85 (27.42) (*n* = 4)	16.58 (23.45) (*n* = 2)
Avg RPE Mid-Session				
All	5.2 (*n* = 20)	6.2 (*n* = 20)	6.4 (*n* = 18)	6.2 (*n* = 19)
<65	3.4 (n = 11)	4.4 (*n* = 11)	4.5 (*n* = 10)	4.9 (*n* = 11)
≥65	5.4 (*n* = 9)	6.1 (*n* = 9)	5.9 (*n* = 8)	6.2 (*n* = 8)
Avg RPE Session End				
All	3.3 (*n* = 20)	3.1 (*n* = 20)	3.5 (*n* = 18)	4.0 (*n* = 19)
<65	1.5 (*n* = 11)	1.4 (*n* = 11)	2.1 (*n* = 11)	1.9 (*n* = 11)
≥65	**3.6 (*n* = 9)**	**3.3 (*n* = 9)**	**3.6 (*n* = 8)**	**4.8 (*n* = 8)**

Bold denotes a significant difference between older adults and younger adults.

## Data Availability

Data are available from the authors upon request.

## Appendix A

**Table A1:** Yoga Sequence.

Pose	Sanskrit Name	Description
Set 1		
Mountain	Tadasana	Standing tall, feet underneath the hips
Lateral Raise, hand over head right side		Right hand over head, leaning to the left
Lateral Raise, hand over head left side		Left hand over head, leaning to the right
Standing Twist right side		Gentle twist through the spine, looking right
Standing Twist left side		Gentle twist through the spine, looking left
Forward Fold	Uttanasana	Hinge at the hips, crown of the head reaching towards the floor. Could be supported with hands on the legs.
Downward Facing Dog	Adho Muka Svanasana	Palms flat on the floor, hips/tailbone pointed up towards the ceiling, feet hip distance apart
Tabletop	Bharmanasana	All fours position on hands and knees
Cat	Marjaryasana	All fours position on hands and knees, with shoulder blades separated and back arched
Cow	Bitilasana	All fours position on hands and knees, with ribs dropping down towards the floor and crown of the head up towards the ceiling
Spinal Balance	Dandayamana Bharmanasana	From tabletop, the opposite hand and foot reach out, keeping the hips and shoulders level with the floor
Tabletop	Bharmanasana	
Childs Pose	Balasana	Knees wider than hip distance apart, big toes together, sink the hips back over the heels
Downward Facing Dog	Adho Muka Svanasana	
Plank	Phalakasana	Hands underneath the shoulders, spine long and core engaged. Can be on knees or with legs lengthened
Low plank	Chaturanga Dandasana	Hands underneath the shoulders, elbows point to the back of the room, and chest is lowering towards the floor.
Cobra	Bhujangasana	From low plank, crown of the head reaching up towards the ceiling, hips down on the floor, spine is lengthening
Downward Facing Dog	Adho Muka Svanasana	
Forward Fold	Uttanasana	
Set 2		
Mountain	Tadasana	
Chair	Utkatasana	Hips sinking back and down, chest lifted, as if you were sitting into a chair
Mountain	Tadasana	
Forward Fold	Uttanasana	
Lunge	Ashta Chandrasana	Front thigh parallel to the floor, back leg extended long (heel lifted) with torso upright
Downward Facing Dog	Adho Muka Svanasana	
Plank	Phalakasana	
Low plank	Chaturanga Dandasana	
Downward Facing Dog	Adho Muka Svanasana	
Lunge	Ashta Chandrasana	
Forward Fold	Uttanasana	
Mountain	Tadasana	
Set 3		
Warrior 1	Virabhadrasana 1	Front thigh parallel to the floor, back leg extended long (heel down) with torso upright and hips/shoulders square to the front
Warrior 2	Virabhadrasana 2	Front thigh parallel to the floor, back leg extended long (heel down) with torso upright and hips/shoulders square to the side
Extended Side Angle	Utthita Parsvakonasana)	Front thigh parallel to the floor, back leg extended long (heel down) with torso on the diagonal, forearm supporting the torso by resting on the front thigh
Reverse Warrior	Viparita Virabahadrasana	Front thigh parallel to the floor, back leg extended long (heel down), front arm is lifting up towards the ceiling and gaze is up towards the ceiling
Lunge	Ashta Chandrasana	
Downward Facing Dog	Adho Muka Svanasana	
Plank	Phalakasana	
Low plank	Chaturanga Dandasana	
Upward Facing Dog		
Downward Facing Dog	Adho Muka Svanasana	
Set 4		
Forward Fold	Uttanasana	
Mountain	Tadasana	
Pyramid Right Side	Parsvottasana	Feet are a stride‘s distance apart, hinge at the hips with the spine long. Can be supported with hands on the front leg.
Warrior 3 Right Side	Virabhadrasana 3	Weight in one foot, opposite foot lifted and pressing towards the back wall. Torso parallel to the floor.
Forward Fold	Uttanasana	
Mountain	Tadasana	
Pyramid Left Side	Parsvottasana	
Warrior 3 Left Side	Virabhadrasana 3	
Forward Fold	Uttanasana	
Mountain	Tadasana	
Eagle, Right Side	Garudasana	Standing on one leg, opposite leg crossed over. Arms are crossed, one elbow below the other and forearms wrapped around so palms are touching
Eagle, Left Side	Garudasana	
Mountain	Tadasana	
Forward Fold	Uttanasana	
Downward Facing Dog	Adho Muka Svanasana	
Tabletop	Bharmanasana	
Reverse Tabletop	Ardha Purvottanasana	Feet flat on the floor, hands underneath shoulders, gaze up towards the ceiling. Hips lifted up off the floor.
Boat	Navasana	Balancing on buttocks in a v-shaped position, with torso and legs lifted on the diagonal
Butterfly	Baddha Konasana	Seated posture with soles of the feet together, knees falling open towards the floor
Revolving Head to Knee, Right Side	Parivrtta Janu Sirsa	Right leg extended, left leg bent in with left foot on the right inner thigh. Left hand reaches up and over towards the right toes
Revolving Head to Knee, Left Side	Parivrtta Janu Sirsa	Left leg extended, right leg bent in with right foot on the left inner thigh. Right hand reaches up and over towards the right toes
Supine Figure 4/ Cradle the Baby, Right side	Supta eka pada galavasana	Lying on the back, left leg extended towards the ceiling, right ankle crossing the left thigh. Legs look like a number “4”
Supine Figure 4/ Cradle the Baby, Left side	Supta eka pada galavasana	Lying on the back, right leg extended towards the ceiling, left ankle crossing the right thigh. Legs look like a number “4”
Supine Spinal Twist, Right Side	Supta Matsyendrasana I	Lying on the back, shoulder blades stay pressed into the floor as the knees twist over to right
Supine Spinal Twist, Left Side	Supta Matsyendrasana I	Lying on the back, shoulder blades stay pressed into the floor as the knees twist over to left
Final Relaxation	Savasana	Typically lying flat on the back, legs extended and arms by the side.
Lay on side, transition to-		
Easy Seated Pose	Sukhasana	Seated on the floor, legs crossed
